# Dragon blood resin ameliorates steroid-induced osteonecrosis of femoral head through osteoclastic pathways

**DOI:** 10.3389/fcell.2023.1202888

**Published:** 2023-08-22

**Authors:** Yuhao Liu, Liang Mo, Hongduo Lu, Yangwenxiang Wei, Jiahao Zhang, Samuel Bennett, Jiake Xu, Chi Zhou, Bin Fang, Zhenqiu Chen

**Affiliations:** ^1^ The First Affiliated Hospital, Guangzhou University of Chinese Medicine, Guangzhou, China; ^2^ School of Biomedical Sciences, University of Western Australia, Perth, WA, Australia; ^3^ Shenzhen institute of Advanced Technology, Chinese Academy of Sciences, Shenzhen, China

**Keywords:** *Dracaena cochinchinensis*, Dragon’s blood resin, osteonecrosis of the femoral head, osteoclast, reactive oxygen species

## Abstract

**Objective:** Dragon’s Blood resin (DBR) is a traditional medicinal substance renowned for its diverse pharmacological effects, which consists of potent anti-inflammatory, antioxidant and angiogenic properties. This study aimed to elucidate its therapeutic mechanism in alleviating steroid-induced osteonecrosis of the femoral head (SIONFH).

**Methods:** Techniques such as SPR and LC-MS were employed to identify and analyze the target proteins of DBR in bone marrow macrophages (BMMs). *In vitro*, BMMs were treated with RANKL and DBR, and TRAcP staining and actin belt staining were utilized to assess osteoclast activity. The inhibitory effects and underlying mechanisms of DBR on osteoclastogenesis and reactive oxygen species (ROS) generation were determined using real-time PCR, western blotting and immunofluorescence staining. An *in vivo* SIONFH rat model was set up to assess the curative impacts of DBR using micro-CT scanning and pathological staining.

**Results:** Bioinformatic tools revealed a pivotal role of osteoclast differentiation in SIONFH. Proteomic analysis identified 164 proteins binding in BMMs. *In vitro* assessments demonstrated that DBR hindered osteoclastogenesis by modulating the expression of specific genes and proteins, along with antioxidant proteins including TRX1 and Glutathione Reductase. Notably, the resin effectively inhibited the expression of crucial proteins, such as the phosphorylation of JNK and the nuclear localization of p65 within the TRAF6/JNK and NFκB signaling pathways. *In vivo* experiments further confirmed that DBR mitigated the onset of SIONFH in rats by curbing osteoclast and ROS activities.

**Conclusion:** These findings underscore the potential of Dragon’s Blood as an effective administration for early-stage SIONFH, shedding light on its therapeutic influence on ROS-mediated osteoclastic signaling pathways.

## 1 Introduction

Osteonecrosis of the femoral head (ONFH) is a progressive, disabling and refractory disorder, without a consensus remedy, which is mainly caused by trauma, excessive administration of glucocorticoids and alcohol abuse, predominantly affecting adolescents and adults (Hines et al., 2021). Among nontraumatic causes, accumulated glucocorticoid dose is the most common, and it has exacerbated the trend of younger onset (Cui et al., 2021). It has been reported that steroid-induced ONFH (SIONFH) results from inadequate blood supply, and simultaneously couples with aberrant bone destruction and regeneration (Liu et al., 2017; Hines et al., 2021), mediated by osteoclasts and osteoblasts.

Bone is a dynamic tissue that constantly undergoes remodeling through bone formation by osteoblasts and bone resorption by osteoclasts (Rochefort et al., 2010). Long-term glucocorticoid use is postulated to influence both osteoclast and osteoblast differentiation and function, resulting in disruption to bone repair (Buckley and Humphrey, 2018). Though osteoclasts are critically important for the removal of necrotic bone, aberrant osteoclast activity readily occurring in bone resorption areas frequently leads to subchondral fracture and bone collapse, which are fatal indications of ONFH progression (Chen et al., 2020). Previous studies have shown that the risk of femoral head collapse at the early-stage could be reduced by anti-resorptive agents which inhibit osteoclastic bone resorption (Kim et al., 2005; Hofstaetter et al., 2009). Osteoclastic bone resorption could therefore affect disease prognosis and outcome in the early stage of SIONFH. In addition, accumulated evidence indicates that the imbalance of bone metabolism in the onset of SIONFH is closely related to oxidative stress (Fan et al., 2021), which seems to affect the bone metabolism microenvironment in a dose-dependent manner under the administration of glucocorticoid (Almeida et al., 2011). Approaches that inhibit oxidative stress appear to have potential therapeutic effects for SIONFH (Jia et al., 2017; Li et al., 2017). Our previous work has also demonstrated that the high oxidative stress occurs following osteoclast hyperactivity and subsequently pathology progression of SIONFH (Chen et al., 2020). Based on this, it attempted to scout for a novel antioxidant agent to prevent or mitigate the progression of SIONFH by inhibiting ROS level and hyperactive osteoclasts, thus paving the way for a new therapy.

Dragon’s blood resin (DBR) a deep red resin, also called Chinese dragon’s blood, which is extracted from stems of *Dracaena cochinchinensis* (Lour.) S.C. Chen (Agavaceae), has been used as a renowned traditional medicine in different cultures of world (Fan et al., 2014). In traditional Chinese medicine, DBR is commonly prescribed to improve circulation, promote tissue regeneration for fractures, sprains, and ulcers, and to control bleeding and pain (Gupta et al., 2008). Modern pharmacological studies have found that this resinous medicine has anti-inflammatory, wound-healing promotion, anti-oxidation, anti-angiogenesis and neural protection (Li et al., 2014; Jura-Morawiec and Tulik, 2016). The components of DBR including loureirin B and dracorhodin have been proved to have suppresses RANKL-induced osteoclastogenesis and ovariectomized osteoporosis via attenuating ROS activities in our previous study (Liu et al., 2019; Liu et al., 2020; Zhang et al., 2022). In view of the important role of osteoclasts and ROS in SIONFH, and the biological effects of DBR, it hypothesized that DBR might suppress oxidative stress and osteoclast activity, thus ameliorating SIONFH, and sought to investigate these hypotheses via bioinformatic analysis, proteomics, and experimental validation approaches.

## 2 Materials and methods

### 2.1 Bioinformatic analyses

A gene expression profile dataset (GSE123568), consisting of serum samples from 30 SIONFH patients and 10 non-SIONFH patients after steroid administration, was downloaded from the Gene Expression Omnibus (http://www.ncbi.nlm.nih.gov/geo). Differentially expressed genes (DEGs) were compared using GEO2R with a |fold change (FC)| ≥ 1, and an adjusted *p*-value below 0.01 was used as the cutoff. The volcano plot was generated using the R studio plot packages.

Furthermore, the keywords “osteoclast differentiation,” “osteoclastogenesis,” “osteoblast differentiation,” and “osteoblastogenesis” were used to search for osteoclastogenesis-related and osteoblastogenesis-related information in the Kyoto Encyclopedia of Genes and Genomes database (KEGG, https://www.genome.jp), PubMed database (https://www.ncbi.nlm.nih.gov/pubmed/), and AmiGO database (http://amigo.geneontology.org/).

Gene set enrichment analysis (GSEA) is a method used to analyze whole-genome expression profile chip data and rank genes based on their differential expression between two samples. The DEGs from GSE123568 were uploaded to WebGestalt’s GSEA tool (http://www.webgestalt.org/) for further analysis.

### 2.2 Proteomics analyses

#### 2.2.1 Surface plasmon resonance (SPR) and liquid chromatography–mass spectrometry (LC–MS) techniques

SPR technology represents an advanced biochemical detection approach that relies on physical optical phenomena. It enables the monitoring of molecular binding processes occurring on the chip surface by precisely measuring the refractive index changes of the gold layer induced by molecular interactions (Jadhav et al., 2020). The SPR and LC-MS techniques employed in this study were previously described (Zhang et al., 2022). In brief, the initial step involved using a light-crosslinked reaction to immobilize the chemical molecules of DBR onto the chip surface. The resin powder was prepared at a working concentration of 10 mg/mL with DMSO, and the resulting resin solution was deposited onto the designated area of the 3D photo-crosslinking sensor chip using a high-throughput array printer. Subsequently, the chip was placed into the SPR Biochip Analysis System. Bone marrow macrophages (BMMs) were then collected from mice and cultured. Following incubation, the cells were lysed prior to SPR analysis. The BMMs sample was subsequently applied and cleaned within the SPR Biochip Analysis System. After completing all tests, the chip was subjected to trypsin hydrolysis. Finally, the LC-MS technique was employed to identify and quantify the protein type and content.

#### 2.2.2 Enrichment analysis and PPI network construction

The Gene Ontology (GO) enrichment and KEGG pathway analysis were performed using the Metascape database (https://www.metascape.org/). Statistical significance was determined at a threshold of *p* < 0.05. To construct the protein-protein interaction (PPI) network, we utilized the String database (https://string-db.org/, Version 11.5).

### 2.3 *In vitro* experiments

#### 2.3.1 Materials and reagents

The resin (Batch No. YPA6A0001) of *D. cochinchinensis* (Lour.) S.C. Chen (Agavaceae) was collected in Yunnan, People’s Republic of China, and commercially supplied by the First Affiliated Hospital of Guangzhou Chinese Medical University (Guangzhou, China). The extraction process of the resin was as follows (see Supplementary Figure S1). Briefly, the pieces of DBR were milled to obtain a uniform powder, which was then extracted using 98% ethanol. After stirring and dispersing for 30 min, the supernatant was collected through a 0.2 µm filter. Finally, the lyophilized powder of DBR was obtained after distillation. To identify the active components, high-performance liquid chromatography (HPLC) analysis was performed on 1 mL samples (see Supplementary Figure S2; Supplementary Table S1).

#### 2.3.2 Osteoclast precursor differentiation and MTS assay for cell viability

The long bones from C57BL/6J female mice were flushed to isolate the BMMs and purified as described (Liu et al., 2020), which was approved by the Animal Ethics Committee of Guangzhou University of Chinese Medicine (No. 20210220008). Cell culture and MTS assay for cell viability methods were as described previously (Liu et al., 2019). Cells were stimulated with 50 ng/mL of RANKL, with different concentrations of DBR for 5 days. Then the cells were stained for tartrate-resistant acid phosphatase (TRAcP) activity assay. A light microscope was used to obtain micrographs of TRAcP-stained cells, and the number and area of osteoclast-like cells (with more than three nuclei) were quantified using ImageJ software. In addition, the influence of compounds on the viability of cells was measured by MTS absorbance at 490 nm using a microplate reader.

#### 2.3.3 Podosome belt staining

The BMMs in optimal condition were seeded into 96-well plates. After overnight adherence, RANKL was introduced to induce their differentiation, and DBR was administered at concentrations of 1 μg/mL or 5 μg/mL for the respective treatments. Once mature osteoclasts were observed in the control group, the cells were fixed using 4% paraformaldehyde for 1 h. Subsequently, the cell membranes were permeabilized with 0.5% Triton X-100 for 1 h, followed by blocking with 10% FBS for 2 h at room temperature. Rhodamine phalloidin was then applied to stain the pseudopodia body bands under dark conditions, while the nuclei of osteoclasts were labeled with DAPI-containing anti-fluorescence quenching solution. The samples were promptly captured using a fluorescence microscope.

#### 2.3.4 p65 fluorescence detection

The BMMs were seeded onto a plate, and on the following day, they were pre-incubated with DBR or ethanol for 1 h. Subsequently, RANKL was added at a concentration of 20 ng/mL to initiate co-culture for 1 h. The medium was then discarded, and the cells were fixed with 4% paraformaldehyde for 1 h. To permeabilize the cell membrane, 0.5% Triton X-100 was added and incubated for 1 h. The cells were then blocked with 10% FBS at room temperature for 2 h. Following this, the cells were incubated overnight at 4°C with the primary antibody targeting P65. A secondary antibody labeled with Cy5 was applied and incubated at room temperature for 1 h. For nuclear staining, the cells were incubated with DAPI-containing anti-fluorescence quenching solution for 30 min. Finally, the cells were immediately observed and photographed using a laser confocal microscope.

#### 2.3.5 RNA extraction and real-time PCR

BMMs were seeded into 6-well plates and cultured with or without DBR for 5 days. Total RNA from cell samples was extracted using Trizol reagent according to the manufacturer’s instructions. We used M-MLV reverse transcriptase and oligo dT primers (Promega) to generate cDNA from RNA samples. To evaluate the efficiency of qPCR, serial dilutions of cDNA were used to generate a standard curve. Then, polymerase chain reaction (PCR) amplification for osteoclast specific sequences was conducted. The cycling parameters for PCR were as below: Holding stage (95°C for 5 min), PCR stage (40 cycles of 95°C for 15 s), and annealing at 60°C for 60 s, Melt curve stage (95°C for 15 s, 60°C for 60 s and 95°C for 15 s). Primers used for qPCR are listed in Table 1. The obtained data were analysed by ΔΔCt method.

**TABLE 1:** Gene primer sequences.

Genes	Forward	Reverse
c-fos	GCGAGCAACTGAGAAGAC	TTGAAACCCGAGAACATC
Mmp9	CGT​GTC​TGG​AGA​TTC​GAC​TTG​A	TTG​GAA​ACT​CAC​ACG​CCA​GA
Ctsk	GGGAGAAAAACCTGAAGC	ATTCTGGGGACTCAGAGC
Acp5	TGTGGCCATCTTTATGCT	GTCATTTCTTTGGGGCTT

#### 2.3.6 Western blot assay

To study osteoclast-specific protein expression, the freshly isolated BMMs were seeded into 6-well plates (1x10^5^ cells per well), and cultured with RANKL stimulation on day 0, 1, 3, and 5 in the presence or absence of DBR (5 μg/mL). To examine signaling pathway proteins, the BMMs were seeded into 6-well plates (2.5 × 10^5^ cells per well) and cultured with culture medium. DBR (5 μg/mL) was used to pretreat the cells for 1 h followed by RANKL stimulation for 0, 5, 10, 20, 30, and 60 min. After the stimulation, radioimmunoprecipitation assay lysis buffer was used to extract total cellular proteins. SDS-PAGE was used to isolate all proteins and transfer the protein bands to a nitrocellulose membrane. After being blocked in 5% skim milk, the membranes were incubated in sequence with primary antibodies (at 4°C for overnight) and secondary antibodies (at room temperature for 1 h). Finally, enhanced chemiluminescence reagents and Image-quant LAS 4000 (GE Healthcare) were used to visualize the protein bands. ImageJ software was used to quantify the band intensities.

### 2.4 *In vivo* experiments

#### 2.4.1 Building the SIONFH rat model

The animal protocol was approved by the Institutional Animal Ethics Committee of Guangzhou University of Chinese Medicine (Number: 20190213001) and conducted in accordance with the European Community guidelines. An SIONFH rat model was established to estimate the intervention efficacy of DBR *in vivo*. In brief, 24 SD rats were randomly divided into three groups (8 rats for each group): a vehicle group, a model group, and a model + DBR (180 mg/kg) group. Imiquimod and Methylprednisolone were used to establish the SIONFH model: Imiquimod (IMI, 30 mg/kg) subcutaneously on day 1 and Methylprednisolone (MPSL, 20 mg/kg) intramuscularly on day 2. All injections were then repeated 4 weeks later. Rats in the vehicle group received the same amount of saline at the same time point with the model group. The day after the first modeling, pharmacological interventions were performed. The rats in model + DBR group were given a DBR treatment by gavage every other day for 6 weeks. The two other groups were administered with the same volume of saline by oral gavage. Rats were humanely euthanized and the femurs were collected after treatment for 6 weeks. In order to visualize the ROS fluorescence signals of Dihydroethidium (DHE), the DHE (20 mg/kg) was subcutaneously injected 24 h prior to sacrifice (Chen et al., 2019). The dosage of rats intragastric administration was calculated according to the dose equivalents between humans and laboratory animals based on ratios of body surface area (Zhang et al., 2021).

#### 2.4.2 Histomorphometric and Micro-CT analysis

Rat femoral head samples were collected at 6 weeks after the establishment of the model. After 48 h of fixation in 4% paraformaldehyde (PFA) and 14 days of decalcification in EDTA (14%, PH = 7.4) (Sigma-Aldrich) at 37°C, the femoral head samples were embedded in paraffin and sectioned. Sections of 5-μm thickness were cut using a Leica RM 2155 Biocut Microtome (Leica Microsystems) and collected onto glass slides. Haematoxylin and eosin (HE) staining and TRAP staining were implemented to visualize the bone microstructures and osteoclasts.

Femurs were collected, soft tissues were dissected, and femoral heads were scanned using a Skyscan Micro-CT instrument (Bruker) with the following parameters: source current of 385 μA, source voltage of 65 kV, pixel size of 9 μm, AI filter of 1.0 mm, and rotation step of 0.4°. The images were reconstructed using NRecon software (Bruker), and the data was analyzed using the CTAn program (Bruker). A refined volume of interest, positioned 0.5 mm above the growth plate and with a height of 0.25 mm, was generated. The subchondral region of interest (ROI) in the trabecular bone was manually determined using a constant threshold range of 50–255. Parameters such as bone volume fraction (BV/TV), trabecular number (Tb.N), trabecular separation (Tb.Sp), and trabecular thickness (Tb.Th) were compared among the three groups.

#### 2.4.3 *In vivo* ROS fluorescence detection

After 48 h of fixation in 4% PFA at 4°C and 4 weeks of decalcification in EDTA (0.5 M, PH = 7.4) at 4°C, the fresh rat femoral heads were cryo-protected by incubating in solutions of 20% sucrose and 2% polyvinylpyrrolidone (PVP) for 24 h at 4°C. Using Tissue-Tek optimum cutting temperature (O.C.T.) compound (Tissue-Tech) to embed the resultant tissues. A thickness of 5-μm sections was obtained and then collected using slides. Slides with frozen sections were left to thaw and air-dry at room temperature for 30 min. Coverslips were mounted onto slides using ProLong Gold Antifade Mountant (Thermo Fisher Scientific) with DAPI to stain nuclei. Then, an A1Si confocal microscope (NIKON) was used to observe the regions of interest with appropriate laser settings (DAPI, 358 nm/461 nm; DHE, 490 nm/590 nm).

### 2.5 Statistical analyses

Statistical analysis was performed with GraphPad Prism 7.0 software. A Student’s t-test was conducted for comparisons between two sets of data, while one-way analysis of variance was used to identify significant differences for multiple comparisons among 3 or more groups of data, and *p* < .05 was considered to indicate statistical significance.

## 3 Results

### 3.1 Bioinformatic analyses of SIONFH

**FIGURE 1:**
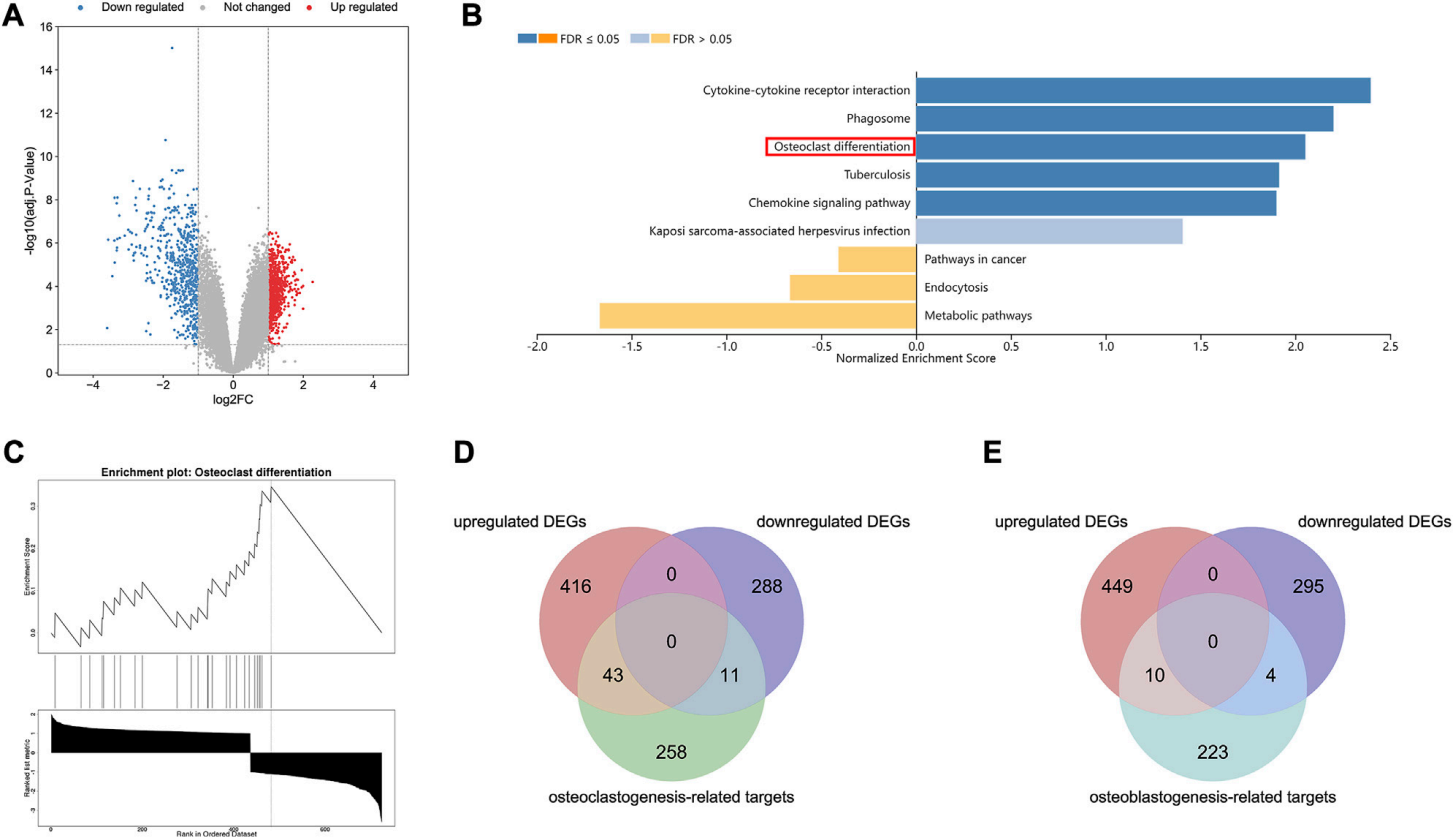
Bioinformatic analyses revealed osteoclastic genes quantitatively dominated in the pathogenesis of SIONFH compared to osteoblast genes. **(A)** Volcano map of the GSE123568, which consisting of 30 SIONFH patients and 10 non-SIONFH patients. The |logFC| > 1 and an adjusted *p*-value <0.05 were set as the threshold to screen differentially expressed genes (DEGs). **(B–C)** Gene Set Enrichment Analysis (GSEA) results of DEGs in GSE123568 using WebGestalt indicated that the osteoclast differentiation was significantly enriched. **(D)** Venn Diagram of intersection between DEGs and osteoclast-related targets. **(E)** Venn Diagram of intersection between DEGs and osteoblast-related targets.

A total of 757 DEGs were identified, consisting of 456 upregulated genes and 301 downregulated genes (Figure 1A). The information of these DEGs in SIONFH samples and normal samples was uploaded to the WebGestalt database for analysis. The GSEA revealed that six gene sets, including osteoclast differentiation, were significantly upregulated in the SIONFH phenotype (Figures 1B,C). Subsequently, 312 gene targets related to osteoclast differentiation and 237 gene targets related to osteoblast differentiation were collected from public databases after removing duplicates. By matching the 757 DEGs with these targets, we identified 54 overlapping targets associated with osteoclastic genes and 14 overlapping targets associated with osteogenic genes in the GSE123568 dataset (Figures 1D,E).

### 3.2 Targets analysis through proteomics

**FIGURE 2:**
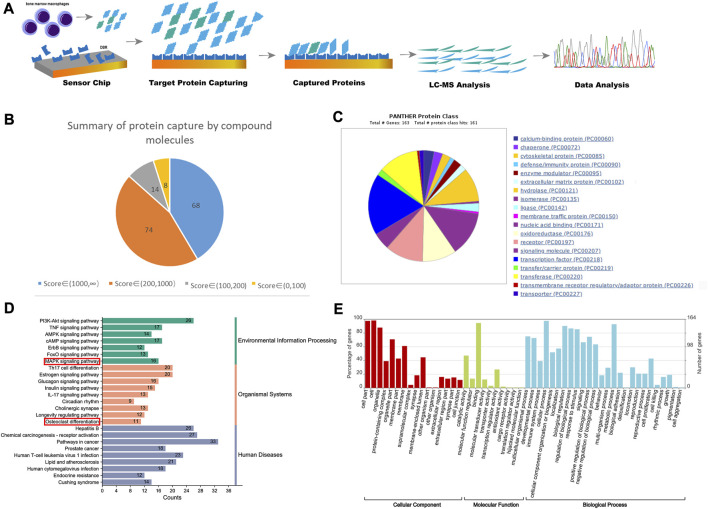
Proteomics detected the binding proteins of DBR in BMMs. **(A)** The diagrams of process and mechanism of the SPR, HPLC and TOF–MS. **(B)** The score of the target protein that DBR acts on BMMs and the proportion of different score segments. **(C)** The protein class hits of DBR targets. **(D)** The KEGG enrichment analysis of DBR targets. **(E)** The enrichment ratios of target proteins in biological process, cell composition and molecular function in biological function obtained by GO enrichment analysis.

Figure 2A illustrates the flowchart of the proteomics analysis conducted. Through SPR, HPLC and TOF–MS, a total of 164 targeted proteins that interact with the resin in BMMs were identified. The scores of these 164 proteins were categorized as follows: 68 proteins had scores above 1,000, 74 proteins had scores ranging from 200 to 1,000, and 22 proteins had scores between 0 and 200 (higher scores indicate stronger affinity and binding speed) (Figure 2B). Figure 2C displays the results of protein classification, indicating that the captured proteins were mainly enriched in transcription factors, signaling molecules, and transferases. The KEGG pathway analysis illustrated the signaling pathways affected by the resin on BMMs, which included pathways involved in cancer, the PI3K-AKT signaling pathway, the estrogen signaling pathway, the MAPK signaling pathway, and osteoclast differentiation (Figure 2D). Furthermore, Figure 2E presents the results of functional enrichment analysis.

### 3.3 Analysis of underlying mechanisms of DBR

**FIGURE 3:**
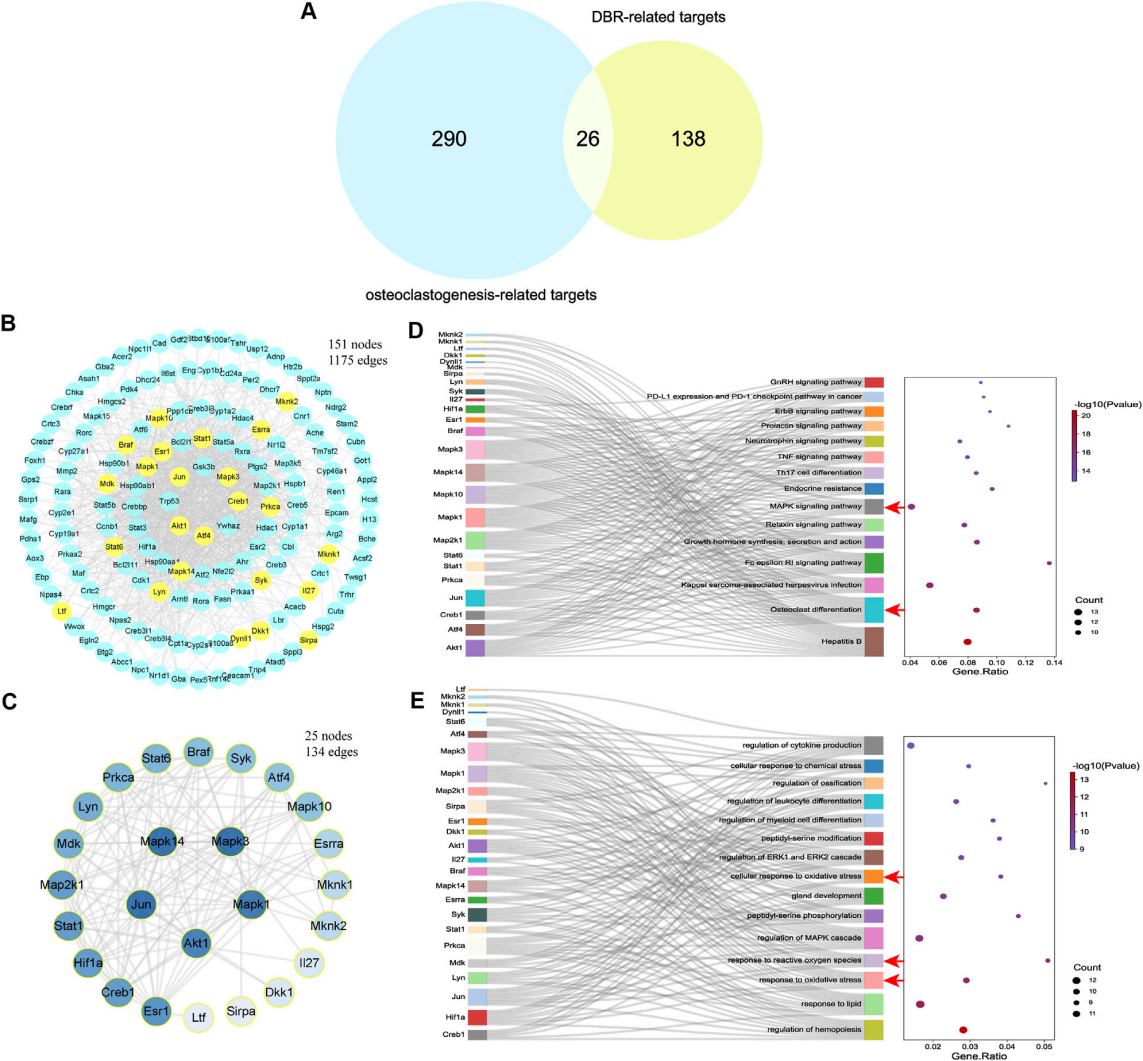
DBR downregulated osteoclast differentiation through MAPK and ROS signaling pathways. **(A)** The Venn diagram presented 26 overlapping targets of DBR and osteoclastogenesis-related targets. **(B)** PPI network of DBR-related targets consisting of 151 nodes and 1,175 edges. Yellow nodes represent overlapping targets with osteoclastogenesis-related targets. **(C)** PPI network of 26 overlapping targets consisting of 25 nodes and 134 edges. **(D)** Sankey diagram for the top 15 KEGG enrichment terms, displayed the relationship between enriched genes and pathway terms. The osteoclast differentiation and MAPK signaling pathway were significantly enriched (red arrows). **(E)** Sankey diagram for the top 15 GO biological process enrichment terms, displayed the relationship between enriched genes and pathway terms. The oxidative stress related terms were also significantly enriched (red arrows).

By comparing the 164 DBR-related targets with 316 gene targets associated with osteoclast differentiation, 26 overlapping targets were identified (Figure 3A). A PPI network was constructed using the STRING database, consisting of 151 nodes and 1,175 edges, representing interactions among the 164 DBR-related targets (Figure 3B). Among these targets, several overlapping ones, such as Jun, Mapk3, and Akt1, showed high correlation with a large number of other targets in the network, suggesting their potential crucial roles in regulating bioprocesses. A subset network comprising the 26 overlapping targets was extracted from the PPI network, which consisted of 25 nodes and 134 edges (Figure 3C). KEGG pathway enrichment analysis of these 26 targets revealed that many of the pathways they were associated with were related to osteoclast differentiation and the MAPK signaling pathway (Figure 3D). Furthermore, GO biological process enrichment analysis of these 26 overlapping targets indicated significant enrichment of functions related to oxidative stress (Figure 3E). These findings suggest that DBR may modulate osteoclast differentiation through the MAPK and ROS signaling pathways.

### 3.4 DBR inhibited RANKL-induced osteoclastogenesis *in vitro*

**FIGURE 4:**
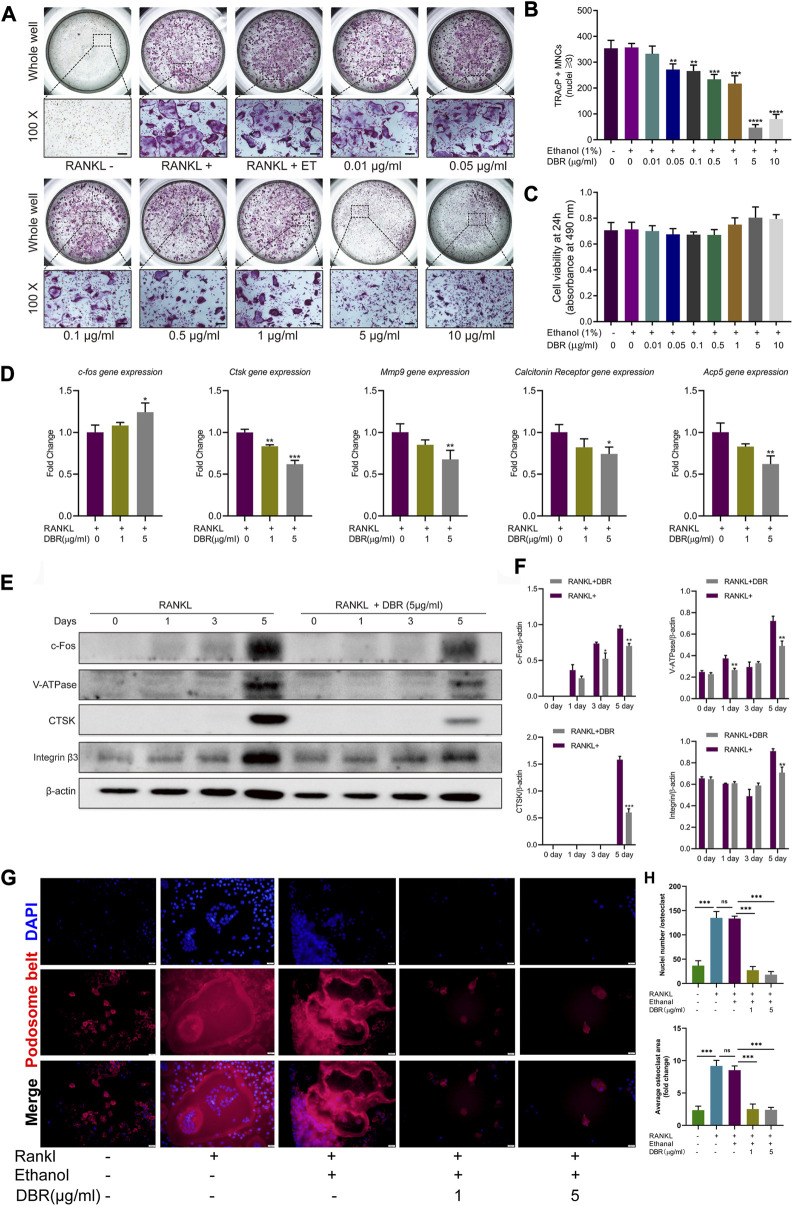
DBR attenuated RANKL-induced osteoclast differentiation and specific osteoclast-related gene expression. **(A)** Representative images showing RANKL-induced osteoclasts treated with DBR at indicated concentrations as examined by TRAcP staining. Scale bar = 200 μm. **(B)** Quantification of TRAcP positive, multinucleated cells (MNCs) in the osteoclast cultures after the treatment of DBR for 5 days. **(C)** MTS assay of the BMMs stimulated with different concentrations of DBR. **(D)** Real-time PCR was utilized to determine the expression levels of osteoclast-specific genes, c-fos, Ctsk, Mmp9, Calcitonin receptor and Acp5. **(E)** Representative images of c-Fos, Integrin β3, V-ATPase and CTSK expression in RANKL-treated osteoclasts after the addition of DBR. Protein expression is normalized to β-actin. **(F)** Quantitative analysis of c-Fos, Integrin β3, V-ATPase and CTSK normalized to β-actin. **(G)** Pseudopodia bands labeled with phalloidin (red) and nuclei labeled with DAPI (blue) after intervention with DBR. Scale bar = 20 μm. **(H)** Quantitative analysis of the area of the pseudopodia band and the number of osteoclast nuclei. All bar graphs are presented as mean ± SD (n = 3). (*p < 0.05, **p < 0.01, ***p < 0.001).

To investigate the effect of the resin on osteoclastogenesis, a standard *in vitro* osteoclast differentiation model was used. The control group, BMM cells were stimulated by RANKL, and exhibited TRAcP-positive multinucleated cells. As demonstrated, the ethanol had no effects on osteoclast differentiation and the reduction of osteoclast formation correlated with increasing concentrations of resin. As little as 0.1 μg/mL resin significantly suppressed RANKL-induced differentiation of BMM cells into osteoclasts (Figures 4A,B). We next evaluated the cytotoxic effects of the resin on BMM cells. According to the result, DBR in the range of experimental concentrations had no significant cytotoxic effect (Figure 4C).

Cells were stimulated with RANKL and intervened with the resin for different time-points. Then, the downstream gene and protein expressions in the osteoclastic signaling pathways were detected using RT-PCR and Western blot. As shown in Figure 4D, RT-PCR showed that DBR increased the level of c-Fos transcripts at Day 5, while the Western blot showed the expression of c-Fos protein was significant inhibited by resin at Day 5, which is associated with mature osteoclast formation. Similarly, the expressions of bone resorption related genes including CTSK, MMP9, and Acp5 were downregulated followed DBR intervention at Day 5. Moreover, the results of Western blot at Day 5 revealed that the expressions of osteoclastic proteins such as c-fos, V-ATPase, CTSK and integrin were downregulated by DBR (Figures 4E,F). Remarkably, fluorescent staining of the pseudopodia band demonstrated that DBR at a concentration of 1 μg/mL and 5 μg/mL effectively impeded the formation of this band, as evidenced by a notable decrease in both the area covered by each osteoclast and the number of nuclei (Figures 4G,H).

### 3.5 DBR attenuated the TRAF6/JNK, NFκB pathways and ROS production

**FIGURE 5:**
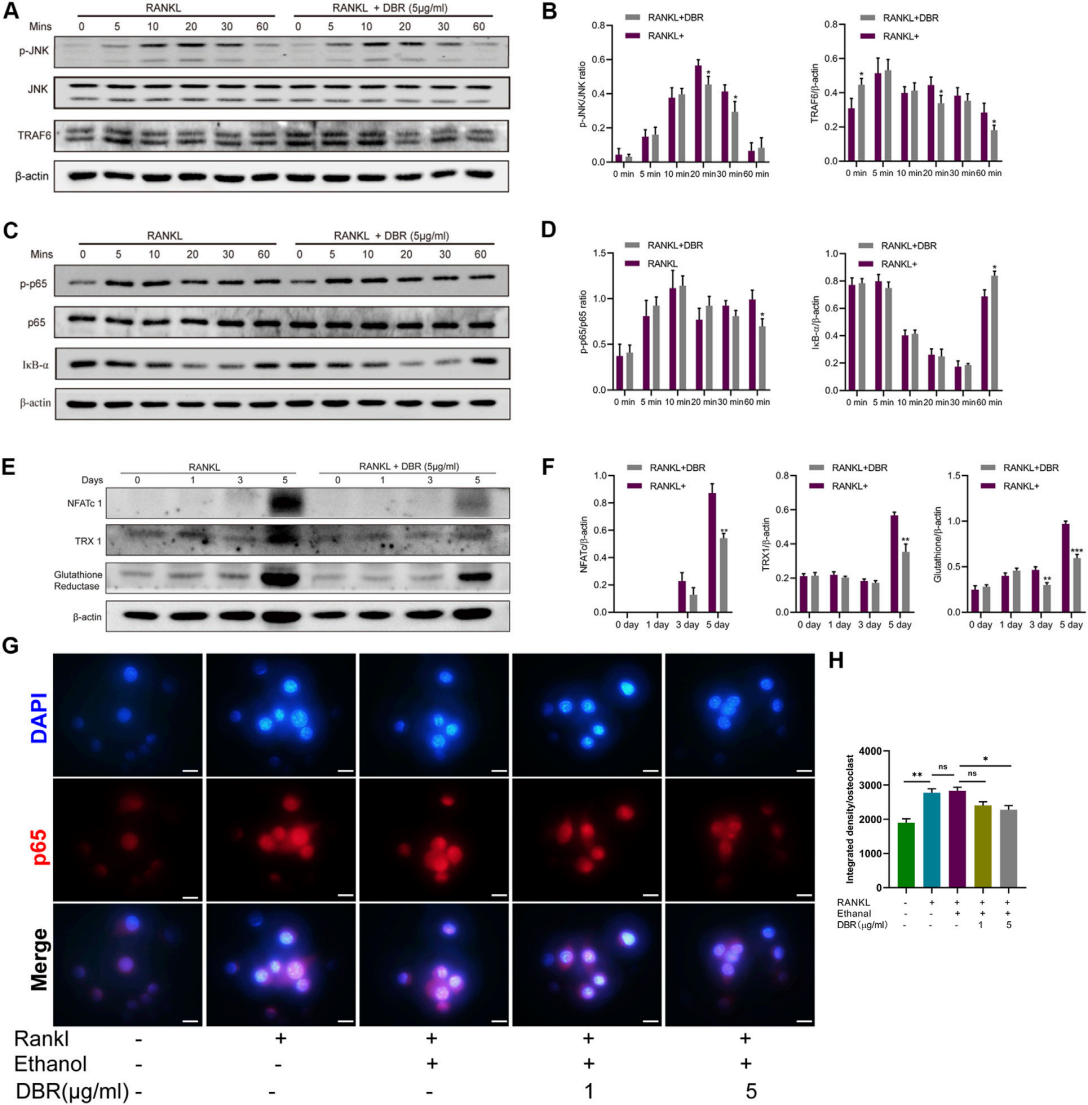
DBR regulated intracellular MAPK and ROS signaling in RANKL-induced osteoclasts. **(A)** Representative images of phosphorylation of JNK and TRAF6. **(B)** Quantitative analysis of phosphorylation of JNK and TRAF6 normalized to JNK and β-actin. **(C)** Representative images of P65 and IκB-α expression in RANKL-treated osteoclasts after the addition of DBR. Protein expression of IκB-α is normalized to β-actin. **(D)** Quantitative analysis of P65 and IκB-α normalized to P65 and β-actin. **(E)** Representative images of NFATc1, TRX1 and glutathione reductase expression. Bone marrow macrophages (BMMs) were pretreated with DBR prior to the stimulation with RANKL. Total protein was analyzed by Western blot at 1, 3, and 5 days. **(F)** Quantitative analysis of protein expression levels of NFATc1, TRX1, and glutathione reductase normalized to β-actin (n = 3). **(G)** Immunofluorescence staining and quantification **(H)** for nuclear localization of p65. Scale bar = 100 μm. All bar graphs are presented as mean ± SD (n = 3). (*p < 0.05, **p < 0.01, ***p < 0.001).

BMMs were stimulated with RANKL, and the expression of TRAF6 and the phosphorylation of MAPK and IκB-α were assessed by Western blotting. We found that DBR treatment suppressed TRAF6 and phosphorylated JNK and kinase (Figures 5A,B). In addition, DBR suppressed the phosphorylation of p65 and delayed the degradation of RANKL-induced IκB-α protein (Figures 5C,D). Shown by the results, the resin strongly suppressed the expression level of NFATc1 protein (Figures 5E,F). In conjunction with the inhibition of DBR on osteoclastic proteins such as c-fos, V-ATPase, CTSK and integrin, the resin regulates osteoclastogenesis by modulating NFATc1 protein expression and, consequently, the expression of NFATc1-driven genes. Taken together, these results indicated that DBR regulates TRAF6/JNK and NFκB pathways, thus influencing osteoclast differentiation. These findings were consistent with the results obtained from fluorescence detection of P65 nuclear migration, indicating the inhibitory effect of DBR on P65 migration into the nucleus (Figures 5G,H).

To investigate the effect of DBR on RANKL-induced intracellular ROS levels during osteoclast differentiation, the expressions of ROS-related proteins, including TRX1 and glutathione reductase, were examined. As shown in Figures 5E,F, the protein levels of TRX1 and glutathione reductase in BMMs were downregulated in the DBR group compared with the control group at day 5. These results suggest that the resin has potent antioxidant properties in BMMs.

### 3.6 DBR ameliorated SIONFH *in vivo*

**FIGURE 6:**
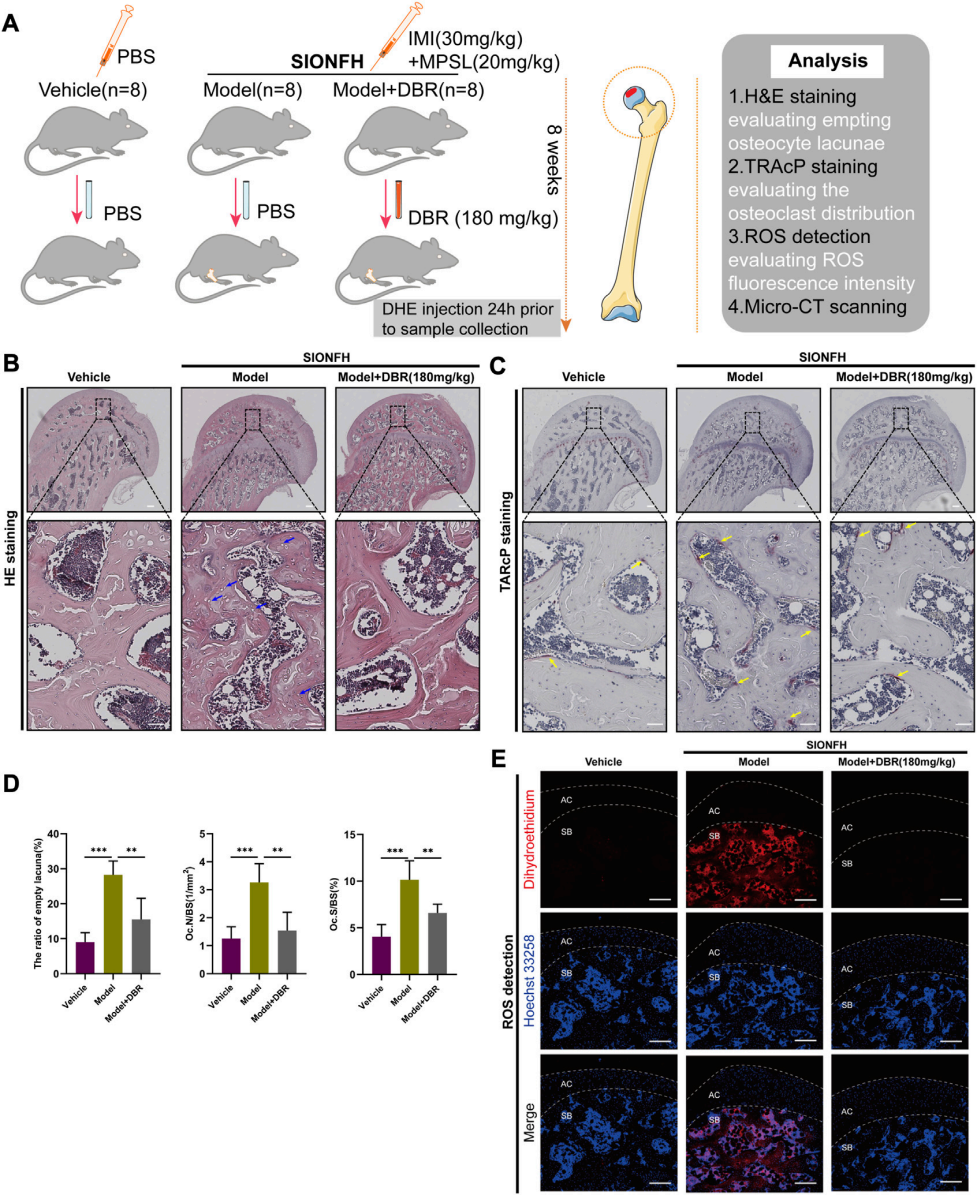
DBR ameliorated SIONFH rats by inhibiting ROS and osteoclast activity. **(A)** Schematic illustration of the establishment of rat ONFH model. All rats were randomly divided into three groups: vehicle group, model group, and model + DBR (180 mg/kg) group. **(B)** Hematoxylin and Eosin (HE) staining of decalcified paraffin-embedding sections showing the increased number of empty lacunae (green arrows) and adipose tissue area (asterisk) in the model group, while the number of empty lacunae was decreased in model + DBR group. **(C)** Tartrate-resistant acid phosphatase (TRAP) staining showing the osteoclasts (yellow arrows) were increased in the model group, but were decreased by DBR. **(D)** Quantitative analyses of the ratio of empty lacuna, N.OC/BS and Oc.S/BS, All bar graphs are presented as mean ± SD (n = 5). N.Oc/BS, osteoclast number/bone surface; Oc.S/BS, osteoclast surface/bone surface. (*p < 0.05, **p < 0.01, ***p < 0.001). **(E)** Cryosections of femoral heads showing ROS level, as probed by dihydroethidium (DHE), in the vehicle group, model group and model + DBR group. AC, articular cartilage (white dashed line area); SB, subchondral bone.

To further explore the effects of DBR on SIONFH, a rat model was used to mimic SIONFH (Figure 6A). From the H&E staining, compared with the vehicle group, the presence of empty osteocyte lacunae was reduced by DBR in the SIONFH rat model. What’s more, TRAcP staining showed the osteoclast number per bone surface and osteoclast surface area per bone surface were decreased after DBR treatment when compared with the non-treatment group. The findings demonstrated that resin suppressed the number of osteoclasts caused by the steroid, indicating that DBR could ameliorate early-stage SIONFH *in vivo* (Figures 6B–D).

The oxidative fluorescent probe DHE and confocal microscopy were used to detect *in situ* levels of ROS in the cryosections of femoral head. As was shown in Figure 6E, DHE fluorescence in the microenvironment of the subchondral area in femoral head was dramatically enhanced following steroid treatment, while it was significantly reduced by DBR treatment in the SIONFH rat model. Collectively, these results demonstrated that DBR treatment could ameliorate early-stage SIONFH through suppressing osteoclast activity and oxidative stress.

### 3.7 Micro-CT based analyses of rat femoral heads

**FIGURE 7:**
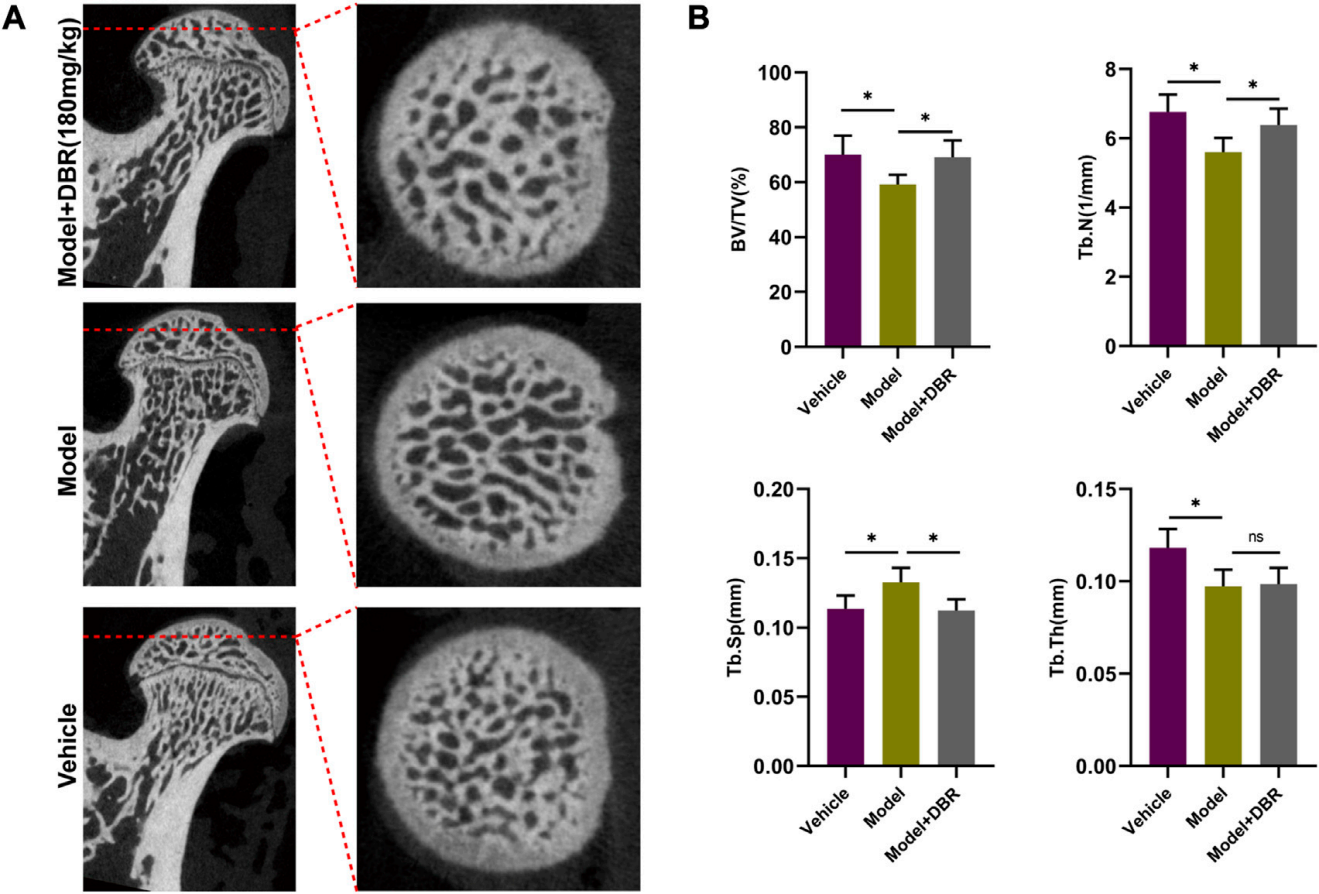
DBR ameliorated SIONFH rats by reducing bone loss. **(A)** Representative micro-CT scanning images of the femoral head. **(B)** Quantification of the bone microstructure parameters of the femoral head, including bone volume/tissue volume (BV/TV), trabecular number (Tb.N), trabecular thickness (Tb.Th) and trabecular separation (Tb.Sp). All bar graphs are presented as mean ± SD (n = 5). (*p < 0.05, **p < 0.01, ***p < 0.001).

To evaluate the subchondral trabecular architecture and bone structural integrity of the femoral head, micro-CT imaging was utilized. As shown in Figure 7A, notable changes in the bone microstructure were observed in the ONFH model group after steroid administration. Furthermore, the analysis of parameters such as BV/TV, Tb.N, and Tb.Sp in the ONFH model group indicated a significant deterioration compared to the control group, suggesting bone loss in the femoral heads of ONFH model rats. However, this trend was significantly improved with DBR treatment (Figure 7B).

## 4 Discussion

ONFH represents the common cause of disability for young adults, whilst the pathogenesis has not yet been fully clarified and the treatments are mainly towards providing symptomatic management (Joint Surgery Group of the Orthopaedic Branch of the Chinese Medical A, 2015). Based on our previous study, ROS level and osteoclast alterations appear to take part in the pathogenesis of SIONFH (Chen et al., 2020). Traditional medicine using DBR has been shown to have pharmacological effects of antioxidation (Jura-Morawiec and Tulik, 2016). However, there is a lack of published studies that provide evidence of the resin’s effectiveness in treating SIONFH by regulating osteoclast differentiation and ROS level. Therefore, the aim of this study was to investigate whether DBR could have a therapeutic effect on SIONFH through osteoclastic pathways.

Firstly, the bioinformatic analyses results of GSE123568 showed osteoclast differentiation was positively correlated with SIONFH and osteoclastogenesis-related genes dominated quantitatively compared with osteogenic genes, which revealed that osteoclast related functions may play an essential role in ONFH. During the progression of ONFH, especially in the early-stage, changes in the mechanical properties and structure of trabecular bone in the femoral head appeared because of bone resorption overactivity (Cao et al., 2015). A prior study demonstrated bisphosphonates had beneficial effects on ONFH, which verified the role of osteoclasts in the collapse of femoral head (Lai et al., 2005). Therefore, inhibiting osteoclast activity could become a direction of strategic therapeutic importance to prevent collapse in early-stage ONFH.

Then, in order to get the potential targets of DBR in BMMs, a proteomics technique was used to screen the potential targets. Proteomics, a high-throughput screening technology, has been used to uncover the causative mechanisms and predictive biomarkers (Suhre et al., 2021).The proteomics results showed that the resin mainly acts on BMMs through 164 captured proteins, and 26 overlapping targets were further obtained by matching with osteoclastogenesis-related targets. Enrichment analysis results of these 26 targets showed that they were mainly related with MAPK signaling pathway and oxidative stress related functions. Therefore, DBR may exert an effect on BMMs for their differentiation into osteoclasts via these pathways.

The MAPK signaling pathway predominantly consists of ERK1/2, p38/MAPK and JNK signaling pathways, which regulate osteoclastogenesis and bone resorption (Wu et al., 2018). JNK is an important regulator of the osteoclast formation process, suppressing the phosphorylation of which could attenuate osteoclastogenesis (Thouverey and Caverzasio, 2015). TRAF6, upstream of MAPK, plays a critical role in RANKL-regulating osteoclast differentiation and activity. TRAF6-knockout mice displayed a defect in NF-κB signaling and consequently developed osteopetrosis (Naito et al., 1999). In this study, Western blot results indicated that DBR attenuated the expression of TRAF6 and the phosphorylation of JNK, downstream of TRAF6, suggesting that DBR can regulate the TRAF6/JNK pathway and possibly attenuate osteoclast differentiation.

As described above, disruption of NF-κB could lead to impaired osteoclast formation and differentiation. Degradation of IκB-α induced by the binding of RANKL to RANK could further phosphorylate p65, which allows NF-κB to translocate into the nucleus and initiate gene transcription (Xu et al., 2009). Western blot results of a detailed time course revealed that the resin could attenuate the NF-κB pathway through preventing IκB-α degradation and p65 phosphorylation in the *in vitro* experiments. NFATc1, an downstream signaling of the JNK pathway, is regarded as a master regulator of osteoclastogenesis (Chun et al., 2020). This study indicated that DBR suppresses the RANKL-stimulated protein levels of NFATc1, which directly regulates osteoclast specific genes, including c-fos, V-ATPase, CTSK and integrin (Takayanagi et al., 2002).

As an upstream signal of the MAPK and NFATc1 pathways, ROS have been shown to be associated with osteoclast proliferation and differentiation in the process of SIONFH (Almeida et al., 2011; Chen et al., 2020), and approaches inhibiting oxidative stress exert potential therapeutic effects on ONFH (Jia et al., 2017). The JNK pathway is one of the crucial signaling pathways activated by ROS. Previous studies showed that ROS can act on TRX and glutaredoxin to activate ASK1, resulting in the induction of JNK (Nygaard et al., 2018). In addition, NFATc1 is known to be upregulated and auto-amplificated by ROS activity (Kim et al., 2010). The current study demonstrated that TRX1 and glutathione reductase, as the ROS-regulating factors, were suppressed by the resin. However, whether the regulation NFATc1 and JNK by DBR is directly dependent on ROS is unclear, which will require further investigation.

Considering the potential effects of DBR *in vitro*, its effects in a SIONFH rat model were further explored. Previous study has indicated that glucocorticoid treatment can lead to an increase in bone cell death and result in an elevated number of empty osteocyte lacunae (Lui et al., 2022). In this study, the rat model was effective in representing the steroid-induced alterations of ROS and osteoclasts before the femoral head collapse in the early-stage SIONFH. H&E analysis revealed that the presence of empty osteocyte lacunae and osteoclast formation were reduced by DBR treatment, which is consistent with the *in vitro* study. Moreover, the ROS fluorescence of the subchondral area of the femoral head was dramatically enhanced in the model group, and was significantly reduced by resin treatment. Lastly, the micro-CT results of the femoral head specimens confirmed that DBR effectively improved the disarray of bone structure in the ONFH group of rats.

In conclusion, this study suggests that DBR might suppress osteoclast differentiation and ROS production to ameliorate SIONFH at an early-stage. Bioinformatic analyses emphasized the important role of osteoclast function in the process of SIONFH, and targets of DBR acting on osteoclast precursors were further obtained by proteomics. *In vitro* experiments confirmed that the resin can inhibit osteoclast formation via suppressing ROS level, TRAF6/JNK and p65 nuclear translocation signaling pathways, which were further verified by an *in vivo* rat model. This study finds that DBR could potentially be used as a therapeutic drug to attenuate the process of SIONFH and indicates the need for further studies to validate these conclusions, with the aim of further developing the potential clinical application of this treatment in humans.

## Data Availability

The original contributions presented in the study are included in the article/Supplementary Material, further inquiries can be directed to the corresponding authors.

## Supplementary material

SUPPLEMENTARY FIGURE S1 The extraction process of resin.

SUPPLEMENTARY FIGURE S2 High-performance liquid chromatogram of DBR at 220 nm. 1:4′,7- dihydroxyflavone; 2: Loureirin D; 3: Dracorhodin; 4: Loureirin C; 5: Loureirin A; 6: Loureirin B; 7: Liquiritigenin.

## Abbreviations

BMMs, bone marrow macrophages; DHA, Dihydroethidium; DBR, Dragon’s Blood resin; DEGs, differentially expressed genes; GSEA, Gene set enrichment analysis; GO, Geno ontology; HE, Haematoxylin and eosin; IMI, imiquimod; KEGG, Kyoto Encyclopedia of Genes and Genomes; LC-MS, Liquid chromatography–mass spectrometry; LC-MS, Liquid chromatography–mass spectrometry; MPSL, Methylprednisolone; ONFH, Osteonecrosis of the femoral head; OC.T., optimum cutting temperature; PVP, polyvinylpyrrolidone; ROS, reactive oxygen species; SIONFH, steroid-induced osteonecrosis of the femoral head; SPR, Surface plasmon resonance; TRAcP, tartrate-resistant acid phosphatase.
