# Supplementary material for: Dragon blood resin ameliorates steroid-induced osteonecrosis of femoral head through osteoclastic pathways
Source: Front Cell Dev Biol. 2023 Aug 22;11:1202888. doi: 10.3389/fcell.2023.1202888 (PMC10477996; doi:10.3389/fcell.2023.1202888)
Supplement: Supplementary file 1 [file Table1.DOCX]

Supplementary Table1: Main active ingredients in DBR

| **No.** | **ingredient** | **PubChem CID** | **Molecular Formula** | **GI absorption** | **Druglikeness** |
| --- | --- | --- | --- | --- | --- |
| 1 | 4',7-Dihydroxyflavone | 5282073 | C_15_H_10_O_4_ | High | 5* |
| 2 | Loureirin D | 13939318 | C_16_H_16_O_5_ | High | 5* |
| 3 | Dracorhodin | 69509 | C_17_H_14_O_3_ | High | 5* |
| 4 | Loureirin C | 14157896 | C_16_H_16_O_4_ | High | 5* |
| 5 | Loureirin A | 5319081 | C_17_H_18_O_4_ | High | 5* |
| 6 | Loureirin B | 189670 | C_18_H_20_O_5_ | High | 5* |
| 7 | Liquiritigenin | 114829 | C_15_H_12_O_4_ | High | 5* |
