# Supplementary figures and images for: Dragon blood resin ameliorates steroid-induced osteonecrosis of femoral head through osteoclastic pathways
Source: Front Cell Dev Biol. 2023 Aug 22;11:1202888. doi: 10.3389/fcell.2023.1202888 (PMC10477996; doi:10.3389/fcell.2023.1202888)

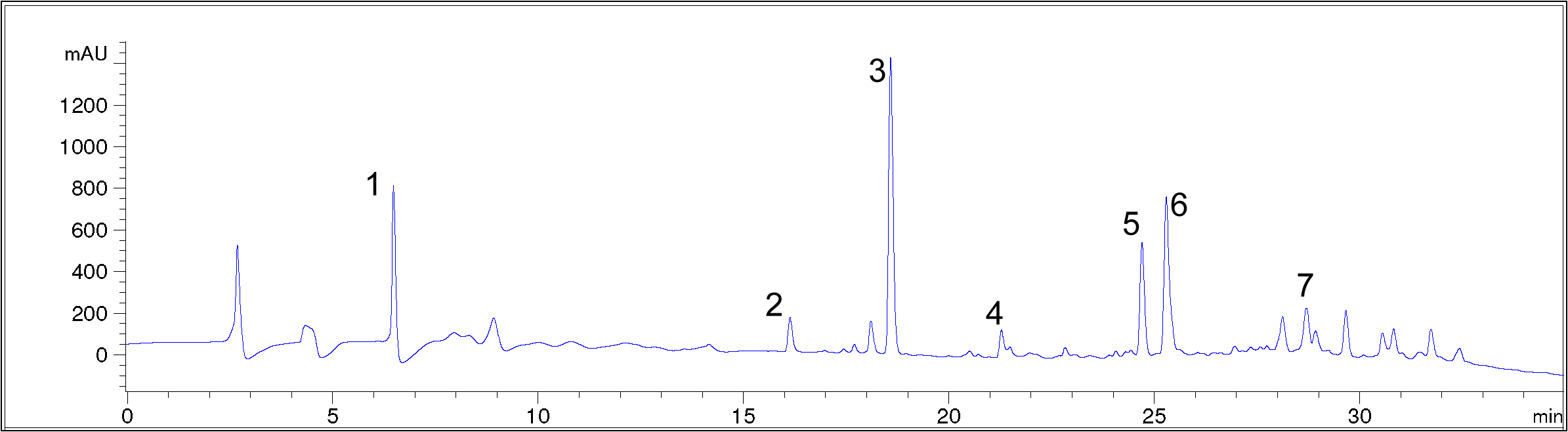

Supplement: Supplementary file 2 [file Image2.TIF]

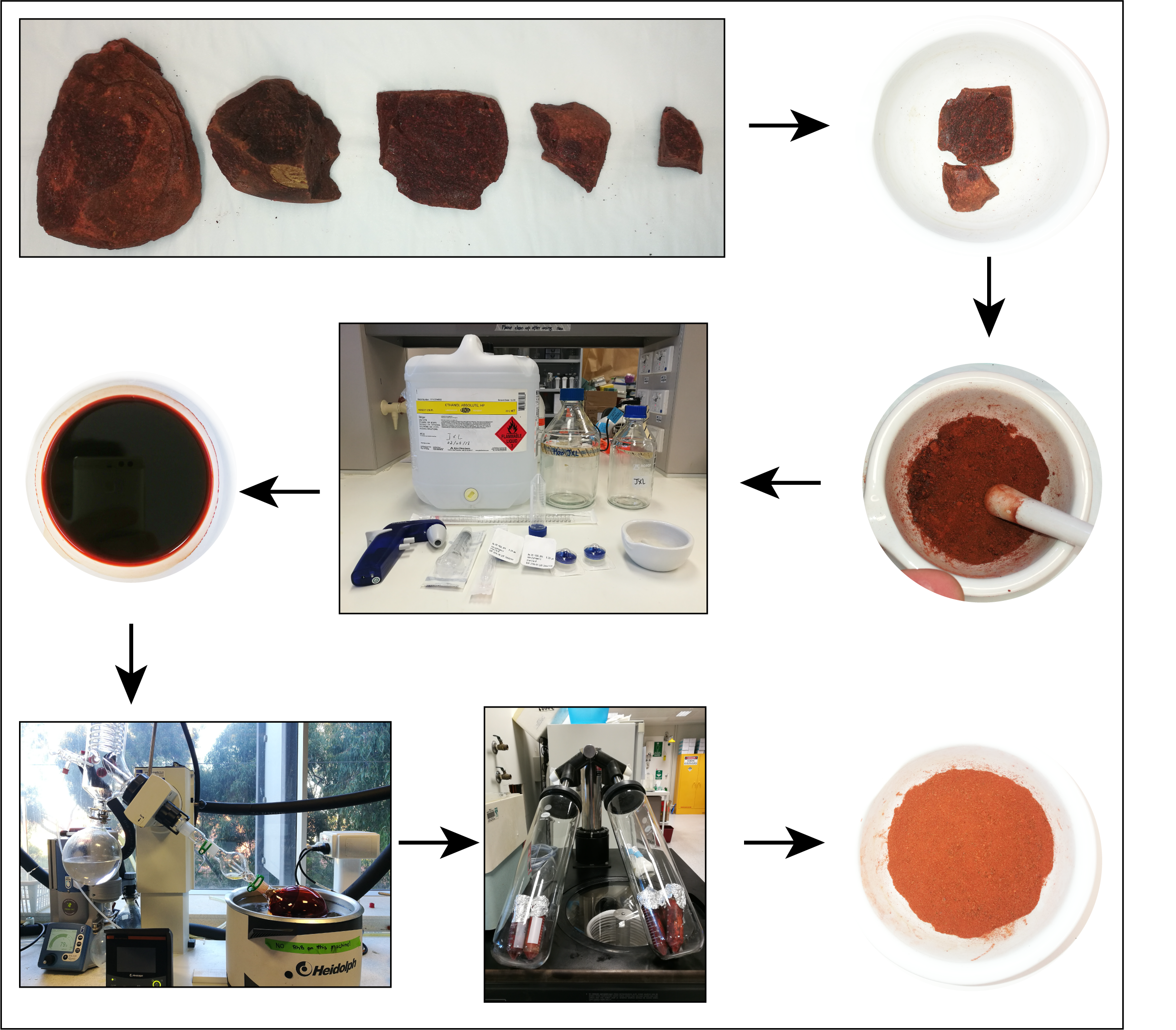

Supplement: Supplementary file 3 [file Image1.TIF]
